# Two Cases of Severe Degeneration of the Macula Following Vitrectomy with Indocyanine Green-Assisted Internal Limiting Membrane Peeling for Idiopathic Macular Hole

**DOI:** 10.2174/1874364100802010027

**Published:** 2008-02-15

**Authors:** Junji Inoue, Toshiro Sakuma, Masatoshi Kiyokawa, Yasuhiko Kobayashi, Hiroshi Takebayashi, Atsushi Mizota, Minoru Tanaka

**Affiliations:** Department of Ophthalmology, Juntendo University Urayasu Hospital, Japan

## Abstract

We describe three eyes of two cases of severe degeneration of the macula following vitrectomy with indocyanine green-assisted internal limiting membrane peeling for idiopathic macular hole. We need to remember the possibility of these complications and have to select the procedures that are safest to use for macular hole surgery.

## INTRODUCTION

Staining the internal limiting membrane (ILM) with indocyanine green (ICG) and peeling it during vitrectomy has greatly improved the rate of macular hole closure [1-3]. This ICG-assisted ILM peeling has become a very common procedure to treat macular holes, although various complications have been reported including visual field defects, retinal tears and detachments, and other alterations of the posterior segment [4-12]. These complications are not always attributable to the ILM peeling or use of ICG, and chorioretinal injuries can be caused by light damage during the surgery and the contact of the retina with the gas used to tamponade the retina [6].

We report two cases of idiopathic macular holes which developed extensive macular degeneration with hyperplasia of the retinal pigment epithelium in the ILM peeled area, although the macular holes were successfully closed.

## CASE REPORT

### Case 1

**Fig. (1):**
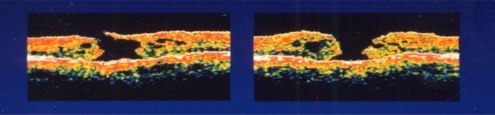
Preoperative optical coherence tomography of both eyes in case 1. Left figure shows right eye (stage III) and right figure is left eye (stage IV).

A 63-year-old woman had a stage III macular hole in the right eye and a stage IV in the left eye (Fig. **1**). She had 6 months history of decreased vision in her right eye and 2 months in her left eye. Her corrected visual acuity was 20/50 OD and 20/30 OS. Phacoemulsification and aspiration, intraocular lens implantation, and vitrectomy were performed on the right eye followed by the left eye. The ILM was stained with 0.5% ICG, and a three to four disc diameter area of ILM was peeled and removed. The initial flap for peeling the ILM was created on the temporal side in each eye. Then 1.0 ml of pure SF_6_ gas was injected to tamponade the retina. The patient maintained a prone position for 24 hours on the first postoperative day and only during the day on the following 7 days.

**Fig. (2):**
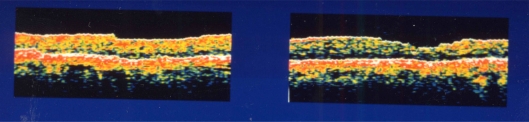
Postoperative optical coherence tomography in case 1. Left figure is the right eye and the right figure is the left eye. Both holes are closed.

**Fig. (3):**
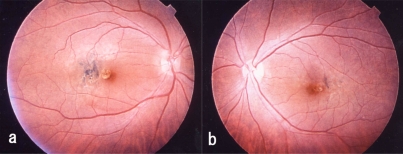
Postoperative fundus photograph of case 1. A yellow round degenerative lesion is seen in the fovea. Irregular degeneration foci associated with pigment deposition can be seen just temporal to the lesion. (**a**, right eye; **b**, left eye).

One week after the surgery, the macular holes were confirmed to be closed (Fig. **2**). However, degenerative changes developed in the macula about 2 weeks after the surgery, especially on the temporal side of the fovea in both eyes. A yellow, round degenerative lesion was noted in the fovea, and irregular degenerative foci associated with pigment deposits were found just temporal to the lesion. The changes were more marked in the right eye (Fig. **3a**,**b**). The surgical procedures and clinical course of left eye was similar to the right eye.

**Fig. (4):**
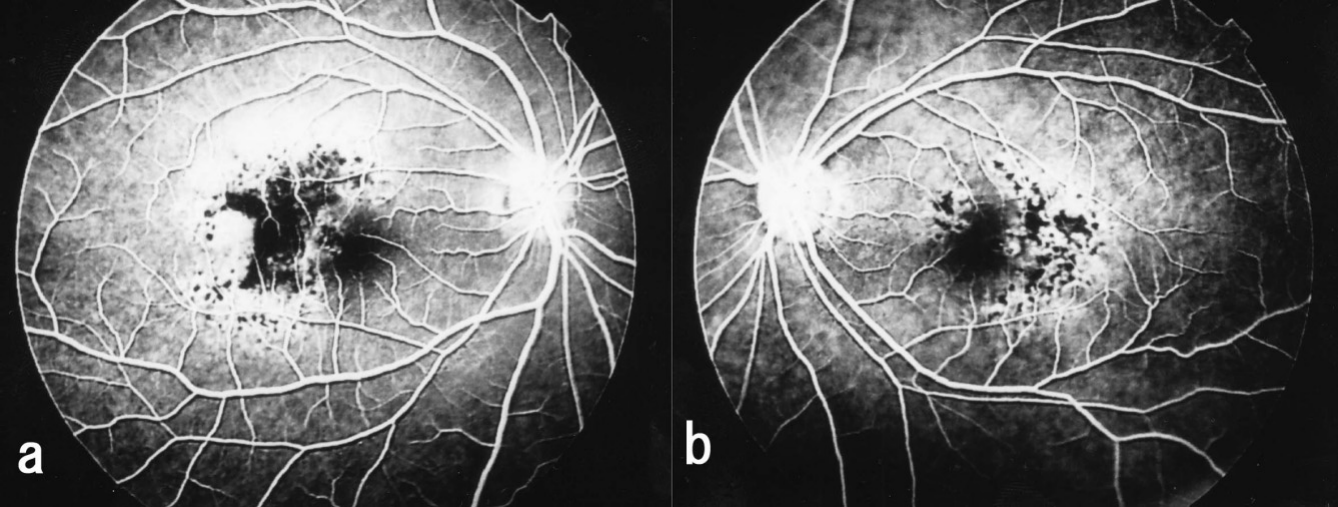
Fluorescein angiogram taken after vitrectomy shows hypofluorescence or blockage of dye in the area with pigment epithelial proliferation in case 1. Hyperfluorescence is noted in the area surrounding the pigment cell proliferation. Many tiny hypofluorescent spots are seen in these areas. (**a**, right eye; **b**, left eye).

Although retinal vessels appeared ophthalmoscopically normal, fluorescein angiography (FA) revealed areas of hypofluorescein and blockage of the fluorescein in the area of the pigment epithelial cell proliferation. The area surrounding the pigment cell proliferation was hyperfluorescein, but tiny hypofluorescein spots were detected within the hyperfluorescein area (Fig. **4a**,**b**).

These pathological changes did not progress, but the visual acuity decreased to 20/60 in the right eye and 20/40 in the left eye at 6 months after surgery.

### Case 2

**Fig. (5):**
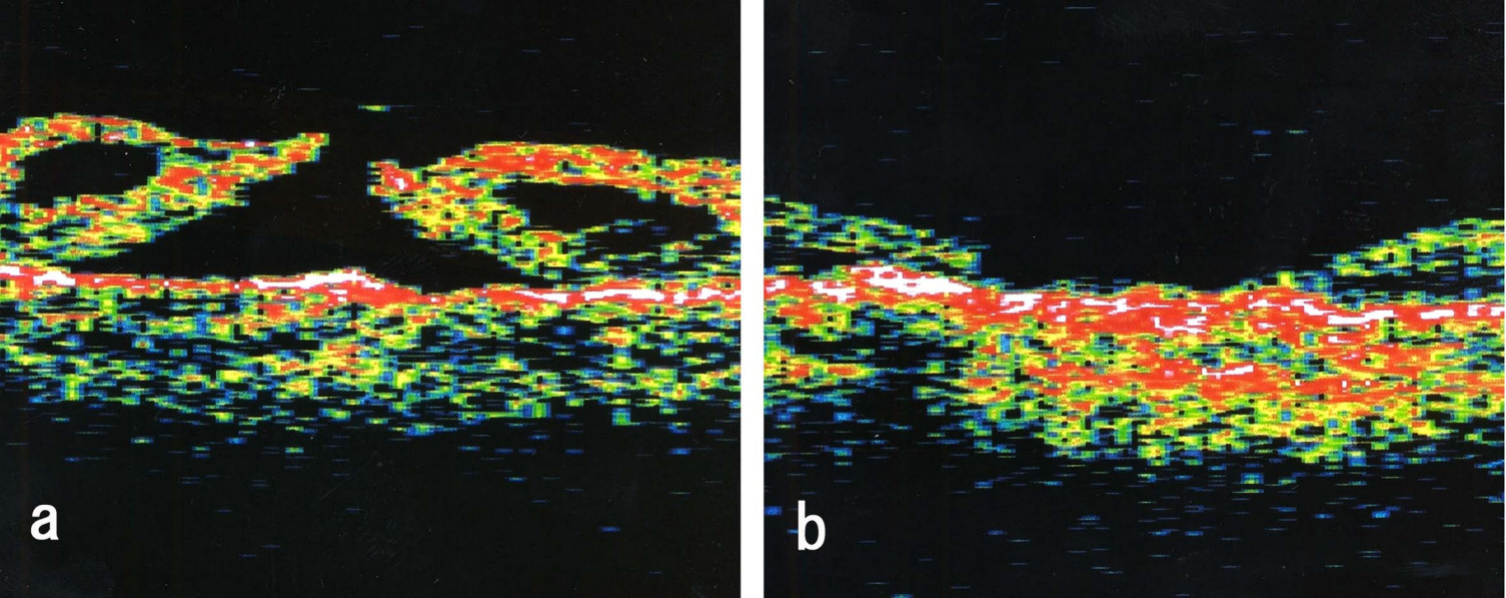
Optical coherence tomograms of left eye of Case 2. **a**: Preoperative optical coherence tomogram showing stage IV macular hole. **b**: Postoperative optical coherence tomogram showing that the macular hole is closed.

**Fig. (6):**
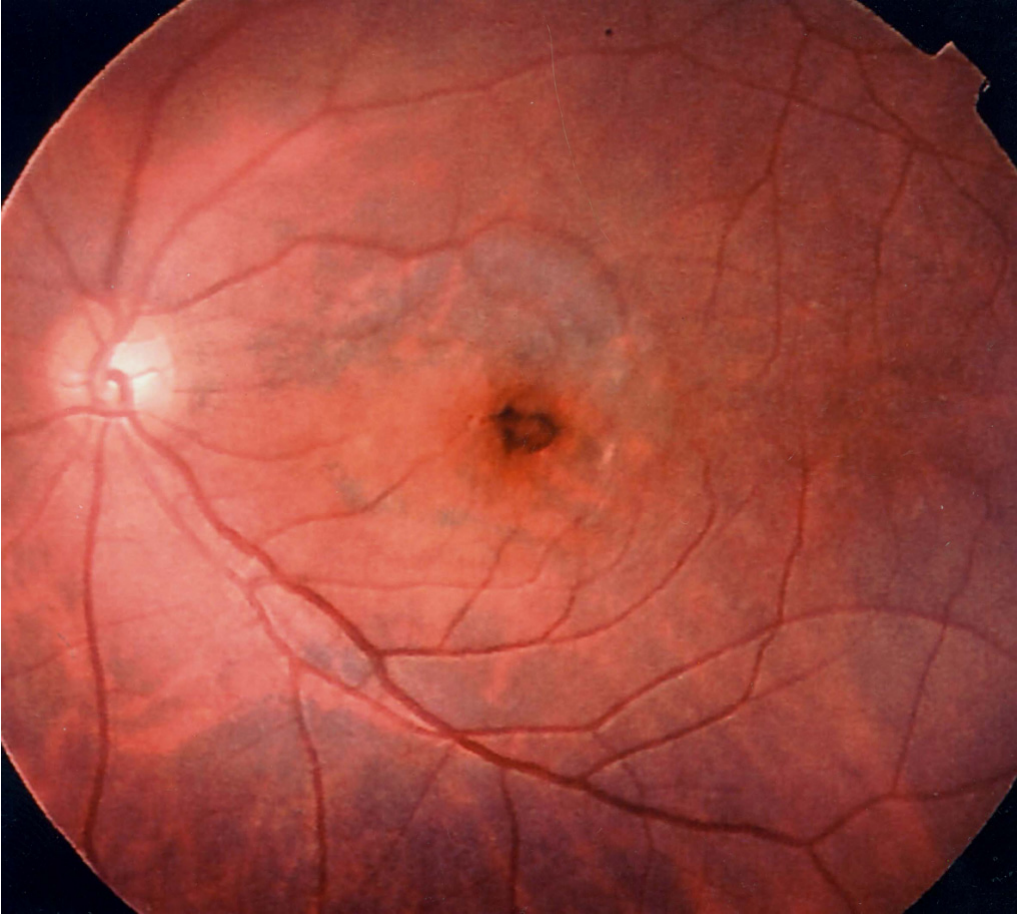
Postoperative fundus photograph showing hyperpigmentation around the macular hole and degeneration of pigment the epithelium within the peeled area of internal limiting membrane in case 2.

**Fig. (7):**
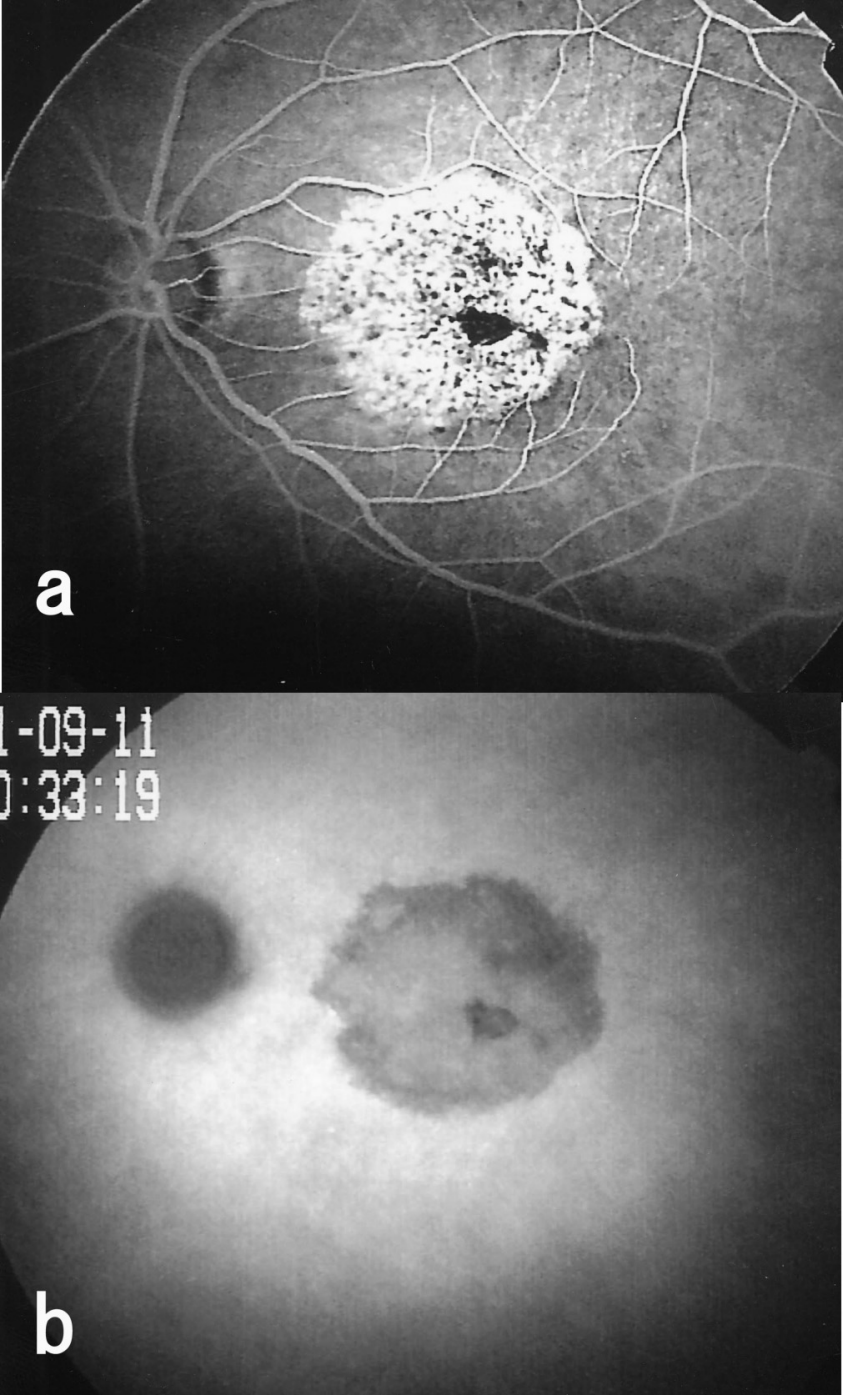
Fluoresceint angiogram and indocyanine green angiogram of the left eye of Case 2. **a**: Postoperative fluorescein angiogram showing hypofluorescence and hyperfluorescence areas. **b**: Postoperative indocyanine green angiogram showing hypofluorescence in the area where ILM was peeled.

A 55 year-old woman had a stage IV macular hole in her left eye (Fig. **5a**). She had 2 months history of decreased vision in her left eye. Her corrected visual acuity was 20/20 OD and 20/60 OS. Phacoemulsification and aspiration, intraocular lens implantation, and vitrectomy were performed. After injecting 0.5% ICG to stain the ILM, a 3 disc diameter area of the ILM was peeled and 0.1 ml of pure SF_6_ gas was injected. The initial flap of ILM was created from the temporal side of the fovea, and it took about one minute to peel the ILM. The patient maintained a prone position on the same schedule as Case 1. The macular hole was closed 1 week after the surgery (Fig. **5b**), however hyperpigmentation developed around macular hole and round degenerative changes were noted within the area of the peeled ILM about 2 weeks after the surgery (Fig. **6**). FA revealed areas of hypofluorescein or a blockage of the fluorescein as seen in Case 1. (Fig. **7a**) Indocyanine green angiography (ICGA) showed hypofluorescein in the area where the ILM was peeled (Fig. **7b**). His visual acuity was 20/40 in the left eye 6 months after the surgery.

Written informed consent was obtained to use ICG during surgery from both cases.

## DISCUSSION

Pigment epithelial cell degeneration in the macula is a relatively rare complication of macular hole surgery with a frequency of 2% to 75% [5-7]. It is generally believed that retinal pigment epithelium damage is caused by surgical trauma during the creation of the initial flap in the ILM and from the abrasive suctioning on the floor of macular hole. Sivalingam *et al*. [13] reported that the visual prognosis was poorer and the recurrence of an epimacular membrane was higher in eyes where ILM components were found in the epimacular membrane removed during vitreous surgery than in eyes where ILM components were not found. It was also reported that postoperative cellular proliferation occurred around the edges of the peeled ILM [14]. Tognetto *et al*. [3] reported that better visual improvement was obtained when the macular hole was closed without ILM peeling. Taken together, these findings suggest that ILM peeling may have some adverse effects on the retina.

ICG has been shown to be toxic to human retinal pigment epithelial cells *in vitro* [15, 16] and in animal experiments [17]. In clinical cases, retinal pigment epithelium atrophy, potentiation of phototxicity, visual field defects, alteration of ILM-retinal cleavage plane by allowing more inner retinal element adherence to the removed ILM, and optic atrophy have been reported after the use of ICG during vitreous surgery [8-12]. Complications in the posterior segment have also been reported after maintaining the prone positioning for a long duration with an expansive gas tamponade [6, 7].

We peeled the ILM, used relatively high concentration of ICG, and used SF_6_. These 3 factors, individually or in combination, might have caused the macular degeneration in our cases. But the contact of the SF_6_ with the macula was for only a relatively short time compared with reported cases in the literature [6, 7] and other cases in our institution. Therefore, the SF_6_ tamponade was most likely not the cause of the macular degeneration in our cases.

ICG staining makes the ILM more visible, and ICG-assisted ILM peeling was performed after creating a posterior vitreous detachment during the macular hole surgery. The ILM was removed easily within one minute because of its visibility. To reduce potential light toxicity, the intraocular fiber optic observation light was directed as far away as possible from the macular hole.

The pattern of macular degeneration in these two cases differed from each other. In Case 1, the pigment proliferation and epithelial cell atrophy developed on the temporal side of the fovea in both eyes. In Case 2, the area of degeneration almost coincided with the ILM peeled area. In Case 1, light damage was considered to be the cause of macular degeneration because degeneration occurred at the site of the initial flap of the ILM was created during surgery. However, illumination on the retina was not excessive in our cases, and the duration of the surgery was similar to other cases without macular degeneration. In Case 2, the area of degeneration roughly coincided with the area of ILM peeling. At the time of ILM peeling, the concentration of ICG in the vitreous must have been very low, but the ICG was in direct contact with the retina without the ILM. In this case, the correspondence of the area of retinal pigment epithelium degeneration and the area of ILM peeled strongly suggest that the degeneration was due to ICG toxicity.

In conclusion, our two cases demonstrated that a degeneration of the retina can occur after ICG-assisted ILM peeling during vitrectomy for a macular hole. The complications are relatively rare but we need to remember the possibility, demonstrated that a degeneration of the retina can occur after ICGassisted ILM peeling during vitrectomy for a macular hole and have to select the procedures that are safest to use for macular hole surgery in each case [2, 18, 19].
